# Novel Poly-Dopamine Adhesive for a Halloysite Nanotube-Ru(bpy)_3_^2+^ Electrochemiluminescent Sensor

**DOI:** 10.1371/journal.pone.0006451

**Published:** 2009-07-30

**Authors:** Bo Xing, Xue-Bo Yin

**Affiliations:** Research Center for Analytical Sciences, College of Chemistry, Nankai University, Tianjin, People's Republic of China; University of Southampron, United Kingdom

## Abstract

Herein, for the first time, the electrochemiluminescent sensor based on Ru(bpy)_3_^2+^-modified electrode using dopamine as an adhesive was successfully developed. After halloysite nanotube slurry was cast on a glassy carbon electrode and dried, an alkaline dopamine solution was added on the electrode surface. Initially, polydopamine belts with dimensions of tens to hundreds of nanometers formed via oxidization of the dopamine by ambient oxygen. As the incubation time increased, the nanobelts became broader and then united with each other to form a polydopamine film. The halloysite nanotubes were embedded within the polydopamine film. The above electrode was soaked in Ru(bpy)_3_^2+^ aqueous solution to adsorb Ru(bpy)_3_^2+^ into the active sites of the halloysite nanotubes via cation-exchange procedure. Through this simple procedure, a Ru(bpy)_3_^2+^-modified electrode was obtained using only 6.25 µg Ru(bpy)_3_^2+^, 15.0 µg dopamine, and 9.0 µg halloysite nanotubes. The electrochemistry and electrochemiluminescence (ECL) of the modified electrode was investigated using tripropylamine (TPA) and nitrilotriacetic acid (NTA) as co-reactants. The different ECL behaviors of the modified electrode using NTA and TPA as well as the contact angle measurements reflected the hydrophilic character of the electrode. The results indicate that halloysite nanotubes have a high loading capacity for Ru(bpy)_3_^2+^ and that dopamine is suitable for the preparation of modified electrodes.

## Introduction

Biomaterials have received extensive interest due to their combination of unique physical and chemical properties. Among these, the proteins secreted by mussels have been of major interest on account of their formation of permanent bioadhesions within the tidal marine environment [1]–[3]. A study on the adhesion mechanism of the secreted proteins indicated that the specialized adhesive protein subtypes contains 3,4-dihydroxy-L-phenylalanine (dopamine) [1]–[3]. By focusing on these properties, dopamine was inserted artificially into some polymer chains to prepare mimic adhesive materials, such as polymers for use as antifouling surfaces [4], [5]. Dopamine modified on Poly(ethylene glycol) (PEG) was used to graft PEG onto solid-state surfaces [4]. Antifouling surfaces using a protein mimetic polymer were also prepared for attaching cells [5]. Xu et al [6] proposed a novel strategy using dopamine as a stable anchor to attach functional molecules on the surface of magnetic nanoparticles. A high-strength bioadhesive analog prepared via layer-by-layer assembly of clay and the dopamine polymer was also successfully developed [7]. Compared with chemical adhesive materials, dopamine-based adhesive or coating is both economical and simple [1], [4]–[7]. In fact, dopamine itself is a good adhesive and coating material [1]. Moreover, if dopamine is directly used as an adhesive, the chemical preparation of dopamine-grafted polymers is unnecessary. Because the adhesive proteins secreted by mussels show a strong adhesion to marine surfaces and dopamine played an important role in the adhesion of mussels as an amino acid contained in these proteins, dopamine-based adhesives are expected to allow binding even under moist conditions or other contaminating environmental conditions [1].

Electrochemiluminescence (ECL) based on Ru(bpy)_3_^2+^ (bpy = 2,2′-bipyridyl) has attracted much research- and application-based interest due to its capacity for detecting a number of analytes [8]–[10]. However, its applications are limited by the consumption of expensive ECL reagents in the solution phase system [8]–[10]. An alternative solution is to immobilize Ru(bpy)_3_^2+^ on solid-state formats for the development of cost-effective, regenerable chemical- or bio-sensors [8]–[10]. Besides reducing the unwanted loss of expensive reagents, this alternative solution has the advantage of an experimental setup that is simplified because no Ru(bpy)_3_^2+^ delivery system is needed [8]–[10].

Ru(bpy)_3_^2+^ adsorbents in combination with anchoring agents can be used to prepare Ru(bpy)_3_^2+^-modified electrodes. Recently, we found that natural halloysite clay nanotubes, while similar to other clay materials [11], [12], can adsorb Ru(bpy)_3_^2+^ via cation-exchange [13]. Moreover, comparing to other clay materials, the tubular structure of these nanotubes appears to impart halloysite materials with a high capacity to adsorb Ru(bpy)_3_^2+^. Herein, the preparation of Ru(bpy)_3_^2+^-modified electrodes using dopamine (3,4-dihydroxyphenethylamine) and the halloysite nanotubes is reported. The preparation, electrochemistry, ECL and hydrophilic property of the Ru(bpy)_3_^2+^-modified electrodes are discussed in detail.

## Results and Discussion

### Preparation of the Ru(bpy)_3_^2+^-modified electrode

**Figure 1:**
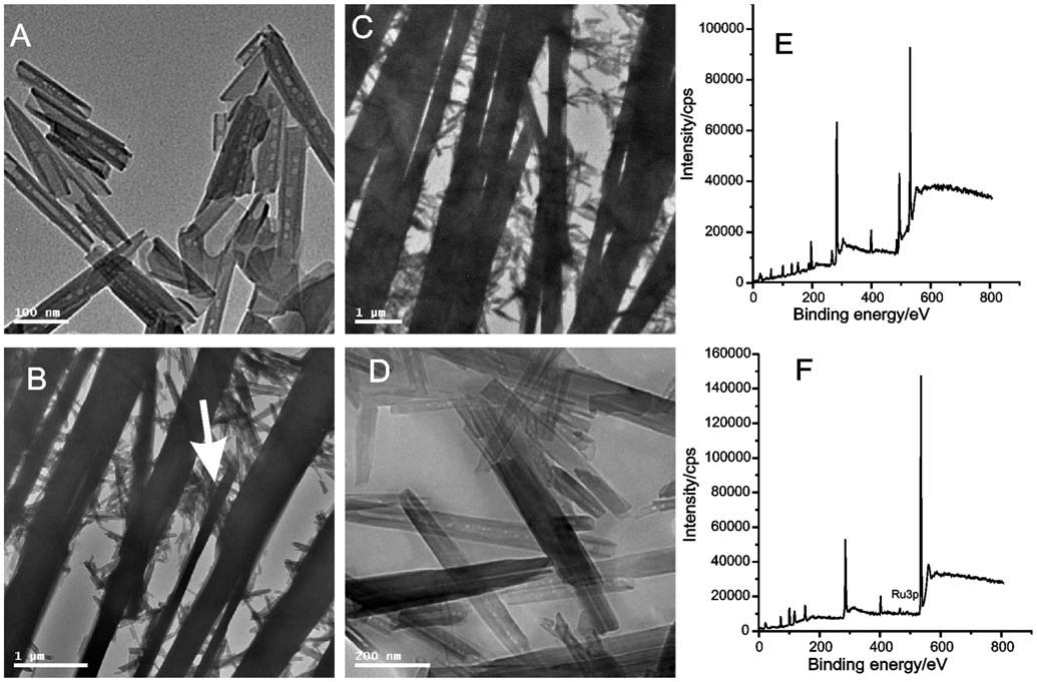
The formation of the polydopamine electrode with embedded halloysite nanotubes. (A) The halloysite nanotubes after being mixed with phosphate buffer solution; (B) 10 min, (C) 15 min, and (D) 1 hour after the dopamine solution was cast on the electrode surface. XPS spectral changes of the polydopamine electrode with embedded halloysite nanotubes before (E) and after (F) adsorption of Ru(bpy)_3_^2+^.

The clay material is characterized as shown in Figure 1A and Figure S1. From Figure 1A, we find that the clay material is in the form of nanometer-sized tubes. Previous works [14] stated that the halloysite nanotubes were geometrically similar to multiwall carbon nanotubes (MWCNTs). But, different to MWCNTs, the halloysite nanotubes observed in Figure 1A are straight without entanglement, which made their dispersion in polymer matrices easy [14]. Figure S1 shows that the X-ray diffraction (XRD) peaks of the nanotubes are consistent with those of halloysite-7A (Al_2_Si_2_O_5_(OH)_4_, JCPDS Card 29-1487). The structure and morphology changes that occurred during the formation of the modified electrode film were observed by transmission electron microscopy (TEM). Aqueous solution which had entered the halloysite nanotubes during mixing afforded the nanotubes with a bean-pod like structure (Figure 1A).

Different to the long formation time of polydopamine films in the bulk solution [1], the formation of aligned nanobelts via oxidation of dopamine just needed 10 minutes in the present work (see Figure 1B). These nanobelts were determined to have dimensions of the order of about ten to several hundred micrometers in length and ten to hundred nanometers in width. Both the previous work [1] and Scheme S1 indicated that dopamine and oxygen are prerequisites for the formation of polydopamine. Under the present conditions, the oxygen in ambient air can participate directly in the oxidation of dopamine and hence accelerate dopamine polymerization, because the dopamine solution forms an aqueous layer on the electrode surface. Therefore, the oxidation of dopamine at the surface is faster than that in the bulk solution [1]. Further, the dimensions of the nanobelts increased, and the nanobelts were found to unite with each other with increasing incubation time, as shown in Figure 1C. After 1 h, the polydopamine film formed completely and the halloysite nanotubes were observed to be embedded within the film. From the data shown in the TEM image (Figure 1D), the thickness of the modified-electrode film was determined to be about 300 nm.

### Dopamine self-polymerization

Although the self-polymerization of dopamine has been extensively used to develop various functional materials [1]–[7], the morphology of polydopamine was observed only in a few works [15]. Just recently, Ouyang et al [15] applied polydopamine nanowires as substrates to imprint protein molecules. polydopamine nanowires formed with an anodic alumina oxide membrane as a nanomold. The effect of the molar ratio of dopamine and ammonium persulfate on the morphology of the polydopamine nanowires was investigated and shown to have a serious effect on their construction [15]. For example, if a molar ratio of 1.5∶1 was used, then the polydopamine grown in the pores of the anodic alumina oxide and formed wall-conglutinated nanotubes. However, the molar ratio of 2∶1 resulted in the formation of nanowires [15]. In the present work, the polydopamine nanobelts were clearly formed and lying in almost the same direction (as indicated by the arrow in Figure 1B). Different to ammonium persulfate used in Ouyang et al's work [15], the ambient oxygen in this work was used as an oxidant for the formation of the polydopamine. Although the molar ratio of dopamine and oxygen was difficult to calculate, the ratio of dopamine and oxygen in the present work may be suitable for the formation of 2-dimensional polydopamine structures. However, because no template was used, the polydopamine grew along the planes of the electrode to form nanobelts. These nanobelts then united with each other to form the polydopamine film.

### Physical characterization of the Ru(bpy)_3_^2+^-modified electrode

Figures 1E and 1F show the X-ray photoelectron spectral (XPS) changes of the polydopamine film comprising embedded halloysite nanotubes before and after adsorption of Ru(bpy)_3_^2+^, respectively. Aluminum (75.5 eV), silicon (104 eV), carbon (284.7 eV), nitrogen (401.5 eV), and oxygen (532.5 eV) photoelectron peaks (in the order of binding energy from low to high) were observed in Figure 1E. The determined area ratio of nitrogen-to-carbon of 0.120 is consistent with that of the theoretical value for dopamine (N/C = 0.125), suggesting that the coating is attributed to polydopamine. The area ratio of silicon–to-aluminum is 1.07, which is similar to that of halloysite (Al_2_Si_2_O_5_(OH)_4_, Si/Al = 1.04). The above results indicate the formation of a polydopamine film with embedded halloysite nanotubes. Besides Al, Si, C, N and O, the ruthenium (463.0 eV) photoelectron peak in Figure 1F validates the adsorption of Ru(bpy)_3_^2+^ on the halloysite nanotubes via ion-exchange. Because the dopamine polymerization was performed under alkaline condition (in 100 mM, pH 8.5 phosphate buffer, prepared with sodium salt), phosphorus and sodium photoelectron peaks were also observed in Figure 1E. Flushing the electrode with distilled water removed not only the non-specifically adsorbed Ru(bpy)_3_^2+^, but also the sodium ions (Figures 1E *cf.* Figure 1F). The Ru(bpy)_3_^2+^ remained on the electrode as confirmed by the preservation of the ruthenium peak in Figure 1F, indicating that Ru(bpy)_3_^2+^ can be specifically adsorbed on the halloysite nanotubes.

From the atomic ratio of silicon-to-ruthenium (Si/Ru = 8.33) shown in Figure 1F, the calculated mass and molar ratio of halloysite nanotubes (based on Al_2_Si_2_O_5_(OH)_4_) and Ru(bpy)_3_^2+^ (based on Ru(bpy)_3_Cl_2_·6H_2_O) are 1.43 and 4.16, respectively, indicating a high adsorption capacity of the halloysite nanotubes for Ru(bpy)_3_^2+^. The mass of adsorbed Ru(bpy)_3_Cl_2_·6H_2_O on a modified electrode was ca 6.25 µg. Compared with the low adsorption capacity of montmorillonite to Ru(bpy)_3_^2+^ [12], the halloysite nanotubes can adsorb much more Ru(bpy)_3_^2+^ due to the tube structure and large area-to-volume ratio. Therefore, only 6.25 µg Ru(bpy)_3_^2+^, 15.0 µg dopamine, and 9.0 µg halloysite nanotubes are deemed necessary for the preparation of Ru(bpy)_3_^2+^-modified electrodes.

### Electrochemical behaviors of the Ru(bpy)_3_^2+^-modified electrode

**Figure 2:**
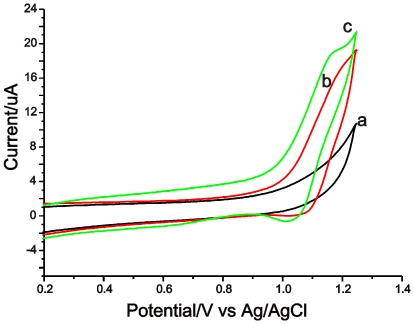
Cyclic voltammograms of the halloysite nanotube-modified electrode in phosphate buffer solution (pH 8.5) without (a) and with (b) 0.5 mM Ru(bpy)_3_^2+^ solution and that of as-prepared Ru(bpy)_3_^2+^-modified electrode (c) in 0.1M phosphate buffer solution (pH 8.5) with a scan rate of 100 mV/s.

The cyclic voltammetry behavior of the Ru(bpy)_3_^2+^-modified electrode can provide important information about the agent transformation, entrapment, activity, and membrane stability. Figures 2a and 2b depict the cyclic voltammograms (CVs) of the polydopamine electrode with embedded halloysite nanotubes in phosphate buffer solution (pH 8.5) with and without 0.5 mM Ru(bpy)_3_^2+^ solution. No redox wave was observed in Figure 2a, showing that the polydopamine film was electrochemically stable under the tested condition. This result is possibly because the dopamine is completely oxidized by ambient oxygen during the formation of polydopamine. Therefore, dopamine as an adhesive material is suitable for the preparation of modified electrodes. When an electrolyte containing 0.5 mM Ru(bpy)_3_^2+^ solution is used, the redox wave of Ru(bpy)_3_^2+^ shows a good transformation of Ru(bpy)_3_^2+^ through the film attached to the electrode surface (Figure 2b). Comparing Figure 2c with Figure 2b, we find the peak current obtained from the Ru(bpy)_3_^2+^-modified electrode is higher than that obtained from the polydopamine electrode comprising embedded halloysite nanotubes in Ru(bpy)_3_^2+^ solution. Meanwhile, the oxidation potential shifts 10 mV in a negative direction possibly due to the reason that no diffusion of Ru(bpy)_3_^2+^ to the Ru(bpy)_3_^2+^-modified electrode surface is necessary. The above results indicated that since the film was approximately 300 nm in thickness and filled with highly-conductive electrolyte, it is much easier for the diffusion of agents and the self-exchange of the electrons through the film.

**Figure 3:**
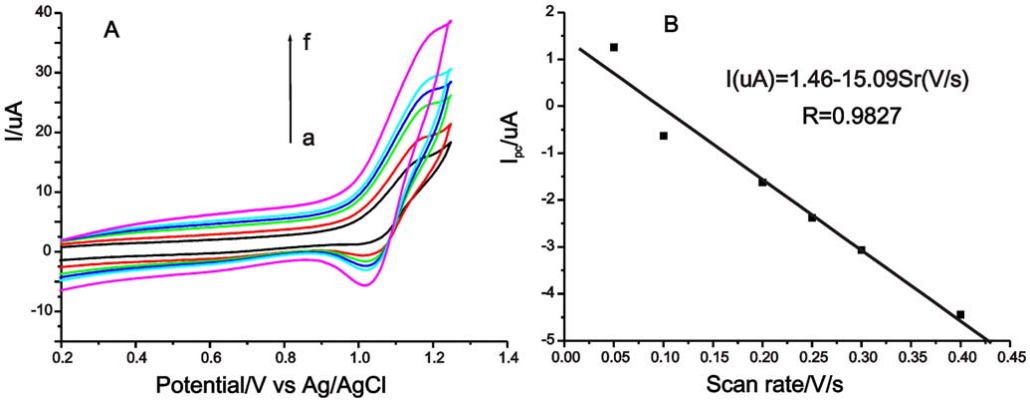
(A) Cyclic Voltammograms of Ru(bpy)_3_^2+^-modified electrode at various scan rates (from inner to outer curve: (a) 50, (b) 100, (c) 200, (d) 250, (e) 300, and (f) 400 mV/s) in 0.1M phosphate buffer solution (pH 8.5). (B) The relationship between the reduction peak currents and the scan rates.

Figure 3 shows the CVs of the as-prepared Ru(bpy)_3_^2+^-modified electrode at various scan rates in 0.1 M phosphate buffer solution (pH 8.5). The observed redox peaks are attributed to the one-electron redox reaction of Ru(bpy)_3_^2+^ [16]–[20]. As shown in Figure 3B, the reduction currents *I_pc_* are directly proportional to the scan rates *v* in the range from 50 to 400 mV/s, indicating that the Ru(bpy)_3_^2+^ electrochemical reaction is a surface-controlled process and Ru(bpy)_3_^2+^ is stably attached on the polydopamine-halloysite nanotube composite film. Moreover, Ru(bpy)_3_^2+^ still retained good electroactivity even though it was bound to the cation sites in the halloysite nanotubes. Hence, the halloysite nanotubes are an effective medium for the adsorption of Ru(bpy)_3_^2+^. The above merits of the modified electrode make it suitable for the development of solid-state ECL sensors.

**Figure 4:**
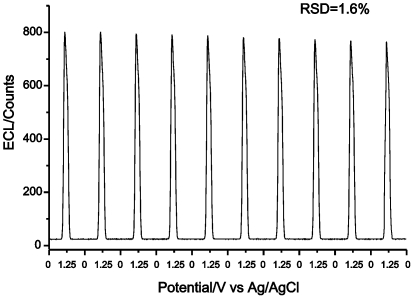
ECL profiles of 0.1 mM TPA in 0.1 M phosphate buffer (pH 8.5) using a Ru(bpy)_3_^2+^-modified electrode under continuous CV for 10 cycles. Scan rate: 100 mV/s.

The peak height from the anodic vs. cathodic scan in the CVs is not but should be consistent to each other because the standard electrochemistry of Ru(bpy)_3_^2+^/ Ru(bpy)_3_^3+^ is a quasi-reversible or reversible procedure. Based on the reproducibilty of the ECL signal (Figure 4) we interpret this as the regeneration of Ru(bpy)_3_^2+^ during the process of ECL emission. As shown in the ECL mechanism (see Supporting Information S1), Ru(bpy)_3_^2+^ is electrochemically oxidized to Ru(bpy)_3_^3+^, which further oxidizes the co-reactant TPA and is reduced to Ru(bpy)_3_^2+^ itself. The formed Ru(bpy)_3_^2+^ is electrochemically oxidized further. In an ECL procedure, the cycle is repeated more than thousand times. This is the reason of the high sensitivity of Ru(bpy)_3_^2+^–based ECL sensor. From the above process, we can find the cathodic current originates only from the reduction of Ru(bpy)_3_^3+^ existing in the system, but the anodic current from the oxidation of Ru(bpy)_3_^2+^ thousands times. The result was similar to the previous works described by Dong's group [18], [19].

### Electrochemiluminescence of the Ru(bpy)_3_^2+^-modified electrode

**Figure 5:**
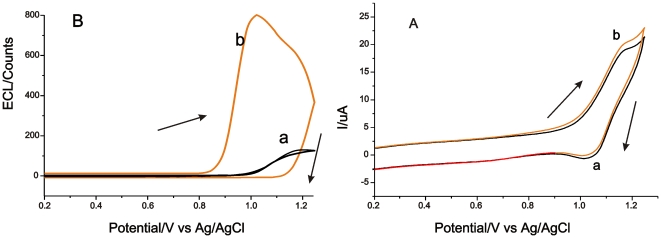
Cyclic Voltammograms (A) and Electrochemiluminescence (B) of Ru(bpy)_3_^2+^ immobilized on the halloysite nanotube modified-electrode with (b) and without (a) TPA (0.1 mM) in 0.1 M phosphate buffer (pH 8.5). Scan rate: 100 mV/s.

The ECL properties of the Ru(bpy)_3_^2+^-modified electrode were tested using tripropylamine (TPA) as the co-reactant. Figure 5 shows the corresponding CV and ECL for the Ru(bpy)_3_^2+^-modified electrode at the scan rate of 100 mV/s in phosphate buffer (pH 8.5) with and without TPA. The CVs of the Ru(bpy)_3_^2+^-modified electrode exhibit a pair of characteristic redox waves of Ru(bpy)_3_^2+^. Moreover, the presence of TPA clearly caused the increase of the oxidation current of Ru(bpy)_3_^2+^ and the decreased reduction current, which was consistent with the Ru(bpy)_3_^2+^-TPA electrocatalytic reaction mechanism [8]–[10]. Meanwhile, the ECL signal increased sharply in the presence of TPA, as shown in Figure 5B. The onset of luminescence was found to occur near 0.9 V, whereafter the ECL intensity rose steeply until it reached a maximum near 1.10 V. The potentials of the onset of luminescence and the maximum potential were lower than those previously reported [21]–[27]. For comparison, if no TPA was present in the electrolyte, then luminescence occurred from about 1.00 V and reached a peak value at 1.18 V with a low emission intensity as shown in Figure 5a.

The previous works [8]–[10] indicated that TPA was oxidized by the electro-generated oxidized form of Ru(bpy)_3_^2+^. However, Bard et al [28], [29] studied the oxidation of TPA and found that the oxidation of TPA at pH values lower than 6.0 was caused by the catalytic homogeneous electron transfer between Ru(bpy)_3_^3+^ and TPA, while the direct oxidation at the electrode surface was possible at pH values higher than 10 [28]. Based on the above discussion, ECL procedures were proposed as shown in Scheme S2 and the ECL mechanism was presented in Supporting Information S1. Here, Ru(bpy)_3_^2+^ is oxidized to form Ru(bpy)_3_^3+^, and the TPA which diffuses into the electrode film is either directly oxidized to generate TPA·radicals on the electrode surface at about 0.8 V or catalytically oxidized by Ru(bpy)_3_^3+^ to form TPA·radicals. The reaction between Ru(bpy)_3_^3+^ and TPA· is found to generate the excited-state Ru(bpy)_3_^2+*^, which emits a photon on relaxation.

Figure S2 shows the relationship between the ECL intensity and the scan rates. The ECL intensity decreased with increasing scan rate over the range of 50–400 mV/s. Similarly, the previous works [19], [30]–[32] illustrated that the Ru(bpy)_ 3_^2+^/TPA system was controlled by intermediate reaction kinetics. The formation of the ECL reactive intermediate and the diffusion of TPA contributed to the variation of the relative ECL intensity with respect to the scan rate as well as the chemical kinetics of the ECL system [19], [30]–[32].

### Hydrophilicity property of the dopamine-based Ru(bpy)_3_^2+^-modified electrode

The Ru(bpy)_3_^2+^-modified electrodes are often used for the purpose of bioarray under aqueous conditions, so the development of a hydrophilic modified electrode is necessary. However, most of the previously developed Ru(bpy)_3_^2+^-modified electrodes, such as those based on Nafion [21]–[23], [32], poly(sodium 4-styrene sulfonate)-silica [33], [34] or benzene sulfonic acid monolayer films [35], are hydrophobic. Moreover, the characteristics of the electrode surface have a significant influence on the ECL emission of the Ru(bpy)_3_^2+^-TPA system [36]–[41]. For example, the hydrophobic electrode surface can concentrate poorly-soluble TPA and hence improve the sensitivity of TPA determination [32], [36]–[41], but it has no such pre-concentration to the soluble co-reactants [32], [36]. The decreased sensitivity toward oxalate relative to TPA is partly due to the lower pre-concentration of oxalate in the hydrophobic modified-electrode film or the slower diffusion in the hydrophobic Nafion-based modified electrode because of the good solubility of oxalate in the aqueous solution [32]. Therefore, the hydrophilicity of an electrode surface can be investigated using the different ECL behaviors of co-reactants with different solubilities [36].

**Figure 6:**
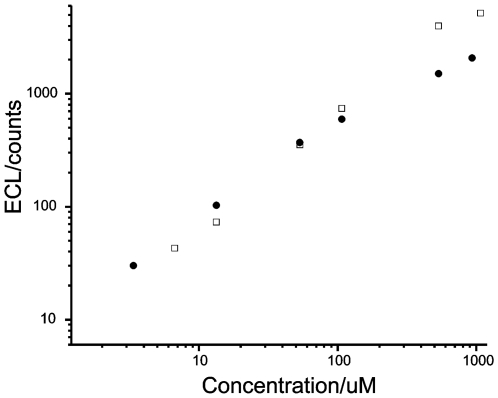
Calibration curves of TPA (□) and nitrilotriacetic acid (•) obtained using a Ru(bpy)_3_^2+^-modified electrode. Scan rate: 100 mV/s.

The co-reactants TPA and nitrilotriacetic acid (NTA), which have different solubilities under alkaline conditions employed in this study, were used to characterize the hydrophilicity of the modified electrode. The dynamic ranges for the ECL intensity *vs* concentrations of TPA and NTA using the Ru(bpy)_3_^2+^-modified electrode were plotted as a log-log profile (Figure 6). It was found that NTA has a higher enhancement on the ECL emission than TPA at low concentrations. The slope of the ECL-concentration profile of TPA is larger than that of NTA. Moreover, the detection limits of NTA are lower than those of TPA using the Ru(bpy)_3_^2+^-modified electrode. At high concentration, TPA and NTA have a similar efficiency to enhance the ECL of Ru(bpy)_3_^2+^.

The above phenomena can be explained as follows: At high concentrations, the higher ECL from TPA is due to its inherently higher excitation efficiency toward Ru(bpy)_3_^2+^ emission, but the diffusion velocity of the two co-reactants is similar because of the high difference in concentration gradient from the bulk solution to the modified electrode surface. However, at low concentration, different to the Nafion-modified electrodes [20]–[22], [32], the present modified electrode has no pre-concentration of TPA but facilitates the diffusion of NTA. Therefore, at their low concentrations, a higher ECL emission was observed with NTA as co-reactant than that with TPA. Based on the above discussions, we conclude that the surface of the as-prepared electrode can be considered as hydrophilic in terms of the ECL behaviors of the modified electrode.

The hydrophilicity of the dopamine-based modified electrode was also characterized by the contact angle measurement. A bare glassy carbon slide without any treatment and the dopamine-halloysite nanotubes-coated glassy carbon slide gave the contact angles of 78.53 and 10.72°, respectively (as shown in Figure S3). The much lower contact angle from the polydopamine-halloysite nanotubes coating indicated the better hydrophilicity of the modified electrode film. Moreover, it is obvious that the good water-compatibility of halloysite and polydopamine results in a hydrophilic Ru(bpy)_3_^2+^–modified electrode. Ouyang et al [15] found the hydrophilicity of the polydopamine material through the contact angle measurements from a pretreated glass slide with polydopamine nanowires, which gave a much lower contact angle, indicating the good hydrophilicity of polydopamine [15].

Figure 4 depicts the ECL signals under continuous cyclic potential scanning for 10 cycles in phosphate buffer solution (pH 8.5) containing 0.1 mM TPA. The RSD (relative standard deviation, n = 10) of the ECL intensity of 1.6 %, suggests the good stability of the ECL determination. Moreover, the modified electrode has good storage stability. If the Ru(bpy)_3_^2+^-modified electrode was stored in the refrigerator (4°C) for one month, then no obvious decrease in ECL intensity was observed with 0.1 mM TPA as co-reactant.

### Conclusion

In conclusion, the self- polymerization of dopamine was used for the first time to prepare a hydrophilic, thin film Ru(bpy)_3_^2+^-modified electrode. Under the present conditions, the dopamine formed first polydopamine nanobelts which then united with each other to form the polydopamine film. In combination of adsorption of halloysite nanotube to Ru(bpy)_3_^2+^, the modified electrode was developed. Different to some of the previously developed Ru(bpy)_3_^2+^-modified electrodes [20]–[22], [32], the present modified electrode showed good hydrophilic property. Dopamine can be applied in the field of modified electrodes as an alternative anchoring agent besides as a target in electrochemistry.

## Materials and Methods

### Instrumentation

The electrochemical measurement of the ECL experiments was carried out using a Model LK98BII Microcomputer-based Electrochemical Analyzer (Tianjin Lanlike High-Tech Company, Tianjin, China). A traditional three-electrode system was employed with Pt wire as the counter electrode, Ag/AgCl/KCl (satd.) as the reference electrode, and a 3 mm-diameter glassy carbon disk as the working electrode. The ECL emission was detected and recorded with a Model MCDR-A Chemiluminescence Analyzer (Xi'an Remax Science & Technology Co. Ltd., Xi'an, China). The voltage of the photomultiplier tube (PMT) in the chemiluminescence analyzer was set at -600 V in the process of detection.

Transmission electron microscopy (TEM) was used to characterize the halloysite nanotubes and confirm the formation of the modified electrode. The crystalline phases of the naturally-occurring halloysite nanotubes were determined by X-ray diffractometry (PANalytical X'PertPRO, Netherlands), using Cu*Ka* radiation. X-ray photoelectron spectra (XPS) were recorded using a Kratos Axis Ultra delay line detector (DLD) spectrometer employing a monochromated Al-*Ka* X-ray source (hv = 1486.6 eV), hybrid (magnetic/electrostatic) optics and a multi-channel plate and DLD. An aperture slot of 300×700 microns was used to record the XPS. Survey spectra were recorded with a pass energy of 160 eV and high resolution spectra were recorded with a pass energy of 40 eV. High-resolution scans were acquired to calculate the chemical compositions of the modified electrode film. The static water contact angle was measured at 25°C by a contact angle meter (JY-82, Beijing Hake Instrumental Company, Beijing, China) using the drop of double-distilled water (DDW).

### Reagents

All the reagents employed were of analytical grade and doubly distilled water was used throughout. Tripropylamine (TPA), dopamine (3,4-dihydroxyphenethylamine), and tris(2,2′-bipyridyl) ruthenium dichloride hexahydrate (Ru(bpy)_3_Cl_2_·6H_2_O) were obtained from Sigma-Aldrich (St. Louis, MO). Nitrilotriacetic acid (NTA) was obtained from The Sixth Tianjin Chemical Company, Tianjin, China. The halloysite materials were kindly donated by Zhengzhou Jinyangguang Chinaware Co. Ltd., Henan, China. 20 mM Ru(bpy)_3_^2+^ solution in DDW as stock solution was stored in a refrigerator prior to use. The working solution was prepared by diluting the stock solutions with phosphate buffer solution (PBS) and then degassed ultrasonically for 10-min immediately prior to use. Sodium dihydrogen phosphate and disodium hydrogen phosphate were used to prepare the electrolyte buffer solution, whose pH was adjusted with 0.1 M NaOH.

### Preparation of dopamine-based Ru(bpy)_3_^2+^-modified electrode

The halloysite clay material is found in its natural state as nanometer-sized tubes, and its XRD peaks (Figure S1) are consistent with those of halloysite-7A (Al_2_Si_2_O_5_(OH)_4_, JCPDS Card 29-1487). Therefore, the clay material is denoted as halloysite nanotubes. To ensure the ion exchange sites of the halloysite clay are in the H-form for adsorption of Ru(bpy)_3_^2+^, the nanotubes were suspended in hydrochloric acid solution (0.1 M) for 10 min. Subsequently, the slurry was thoroughly washed with DDW until the pH of the water became close to neutral.

**Figure 7:**
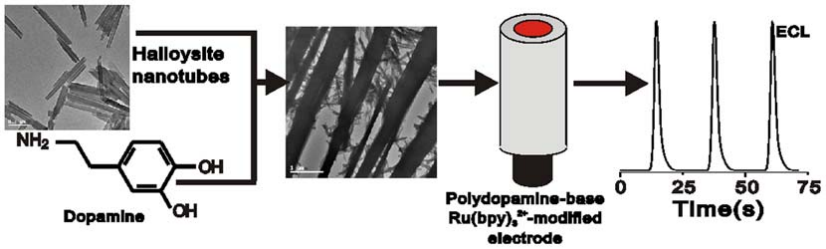
The protocol for the preparation of the Ru(bpy)_3_^2+^-modified electrode.

The protocol for the preparation of Ru(bpy)_3_^2+^-modified electrodes is shown in Figure 7. Before modification, the glassy carbon electrode (GCE) was successively polished with 0.3- and 0.05-µm aluminum slurries and sonicated in firstly ethanol and then DDW. To immobilize the halloysite nanotubes on the electrode surface, 3 mg of as-prepared halloysite nanotubes were added to 1 ml DDW followed by sonicating the aqueous nanotube solution for 10 min. After, 3 µl of the aqueous nanotube slurry was cast on the surface of the GCE and dried at room temperature. 3 µl of 5 mg mL^−1^ dopamine solution in 100 mM phosphate buffer (pH 8.5) was cast onto the electrode surface containing halloysite nanotubes. A polydopamine film was formed on the electrode at room temperature and the halloysite nanotubes were observed to be embedded into the film. The Ru(bpy)_3_^2+^-modified electrode was prepared via soaking the polydopamine-electrode with embedded halloysite nanotubes in an unstirred 0.1 mM Ru(bpy)_3_^2+^ aqueous solution for 2 h. Ru(bpy)_3_^2+^ was adsorbed onto the active sites of the halloysite nanotubes via cation-exchange procedure. Before each experiment, the Ru(bpy)_3_^2+^-modified electrode was rinsed thoroughly with DDW and cyclically swept over the potential range from 0 to +1.25 V in phosphate buffer solution (0.1 M pH 8.5) to remove any non-specifically adsorbed Ru(bpy)_3_^2+^.

Sample for TEM examination were made as followed. 3 µl of the aqueous nanotube slurry was cast on a carbon-coated copper grid and dried. Then, 3 µl of 5 mg mL^−1^ dopamine solution was placed on the copper grid for dopamine self-polymerization. To control different incubation time, the grid containing the solution was nitrogen air-dried after a period of time. Once the solution is dried, the self-polymerization of dopamine ceases because the self-polymerization occurs at alkaline solution.

## Supporting Information

Supporting Information S1 Supporting information available: The treatment of halloysite nanotubes, XRD pattern (Figure S1) of the halloysite nanotubes, the effect of the scan rates on ECL intensity in phosphate buffer solution (pH 8.5) containing 0.1 mM TPA (Figure S2), the contact angles of the bare glassy carbon slide and the polydopamine-halloysite nanotube coated glassy carbon slide (Figure S3), the possible polymerization mechanism of dopamine (Scheme S1), and the possible electrochemiluminescence (ECL) mechanism of the Ru(bpy)32+-modified electrode using tripropylamine (TPA) as a coreactant (Scheme S2).
(0.05 MB DOC)

Figure S1 The XRD pattern of the halloysite nanotubes.
(0.19 MB TIF)

Figure S2 Effect of the scan rates on ECL intensity in phosphate buffer solution (pH 8.5) containing 0.1 mM TPA using the Ru(bpy)_3_^2+^.
(0.08 MB TIF)

Figure S3 The contact angles of the bare glassy carbon slide (A) and the polydopamine-halloysite nanotube coated glassy carbon slide (B).
(4.18 MB TIF)

Scheme S1 Possible structural evolution and polymerization mechanism of dopamine [1].
(0.24 MB TIF)

Scheme S2 The schematic electrochemiluminescence mechanism of TPA in Ru(bpy)_3_^2+^-modified electrode [2], [3].
(1.06 MB TIF)
