# Supplementary material for: Novel Poly-Dopamine Adhesive for a Halloysite Nanotube-Ru(bpy)3 2+ Electrochemiluminescent Sensor
Source: PLoS One. 2009 Jul 30;4(7):e6451. doi: 10.1371/journal.pone.0006451 (PMC2714183; doi:10.1371/journal.pone.0006451)
Supplement: Supporting Information S1 — Supporting information available: The treatment of halloysite nanotubes, XRD pattern (Figure S1) of the halloysite nanotubes, the effect of the scan rates on ECL intensity in phosphate buffer solution (pH 8.5) containing 0.1 mM TPA (Figure S2), the contact angles of the bare glassy carbon slide and the polydopamine-halloysite nanotube coated glassy carbon slide (Figure S3), the possible polymerization mechanism of dopamine (Scheme S1), and the possible electrochemiluminescence (ECL) mechanism of the Ru(bpy)32+-modified electrode using tripropylamine (TPA) as a coreactant (Scheme S2). (0.05 MB DOC) [file pone.0006451.s001.doc]

Supporting Information S1 for

Novel poly-dopamine adhesive for a halloysite nanotube-Ru(bpy)_3_^2+^ electrochemiluminescent sensor

Bo Xing, Xue-Bo Yin*

Research Center for Analytical Sciences,
College of Chemistry, Nankai University, Tianjin, 300071, P. R. China

CORRESPONDING AUTHOR: Dr. Xue-Bo Yin, Research Center for Analytical Sciences, College of Chemistry, Nankai University, Tianjin, 300071, P. R. China, E-mail: xbyin@nankai.edu.cn, Fax: 86-22-23502458

**Content**

**Treatment and characterization of the halloysite mine;**

**Figure S1-S3.**

**Scheme S1-S2;**

**Treatment and characterization of the halloysite mine.** The original halloysite mine is stone-like and rigid. But once it is soaked in water, the particles are dispersed. After being shaken and left for 30 min to sediment, the upper part of the suspension is collected and the rough precipitate is removed. The cloudy suspension is the halloysite nanotubes, which are characterized as shown in Figure 1A and S1. The XRD pattern of the naturally-occurred material is given in Figure S1, where distinct XRD peaks of halloysite-7A (Al_2_Si_2_O_5_(OH)_4_, JCPDS Card 29-1487) appears. TEM photo (Figure 1A) shows the halloysite material has a tubular morphography. The inner and outer diameters are about 20 nm and 40 nm, respectively. The length of the nanotubes is from 200 to 600 nm.

The mechanism governing ECL in the present electrode was proposed as followed [2,3]:

$TPA\text{-}e\to TPA^{+\bullet}\to TPA(-H)^{\bullet}+H^{+}$ (1)

$Ru(bpy)_{3}^{2+}-e^{-}\to Ru(bpy)_{3}^{3+}$ (2)

$TPA(-H)^{\bullet}+Ru(bpy)_{3}^{3+}\to Ru(bpy)_{3}^{2+*}+TPA(-H)^{\bullet+}$ (3)

$Ru(bpy)_{3}^{2+*}\to Ru(bpy)_{3}^{2+}+hv$ (4)

**Reference**

(1) Lee H, Dellatore SM, Miller WM, Messersmith PB (2007) Mussel-inspired surface chemistry for multifunctional coatings. Science 318: 426-430.

(2) Miao WJ (2008) Electrochemiluminescence. Chem. Rev. 108: 2506-2553.

(3) Miao WJ, Choi JP, Bard AJ (2003) Electrogenerated chemiluminescence 69: The tris(2,2'-bipyridine)ruthenium(II) /tri-n-propylamine (TPrA) system revisited - A new route involving TPrA• cation radicals. J. Am. Chem. Soc. 124: 14478-14485.

Figure and Scheme Caption

**Figure S1.** The XRD pattern of the halloysite nanotubes.

**Figure S2.** Effect of the scan rates on ECL intensity in phosphate buffer solution (pH 8.5) containing 0.1 mM TPA.

**Figure S3.** The contact angles of the bare glassy carbon slide (A) and the polydopamine-halloysite nanotube coated glassy carbon slide (B).

**Scheme S1.** Possible structural evolution and polymerization mechanism of dopamine [1].

Scheme S2. The schematic electrochemiluminescence mechanism of TPA in Ru(bpy)_3_^2+^-modified electrode [2, 3].
