# Supplementary figures and images for: Novel Poly-Dopamine Adhesive for a Halloysite Nanotube-Ru(bpy)3 2+ Electrochemiluminescent Sensor
Source: PLoS One. 2009 Jul 30;4(7):e6451. doi: 10.1371/journal.pone.0006451 (PMC2714183; doi:10.1371/journal.pone.0006451)

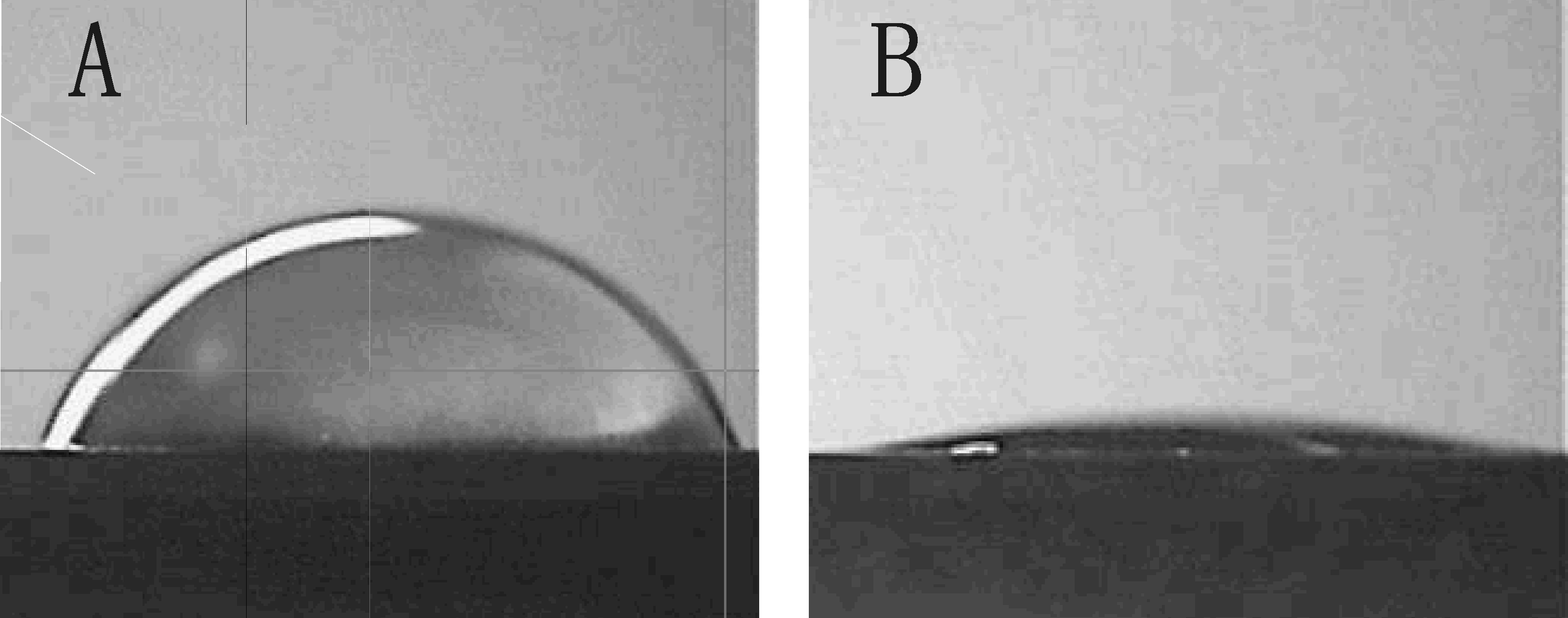

Supplement: Figure S3 — The contact angles of the bare glassy carbon slide (A) and the polydopamine-halloysite nanotube coated glassy carbon slide (B). (4.18 MB TIF) [file pone.0006451.s004.tif]

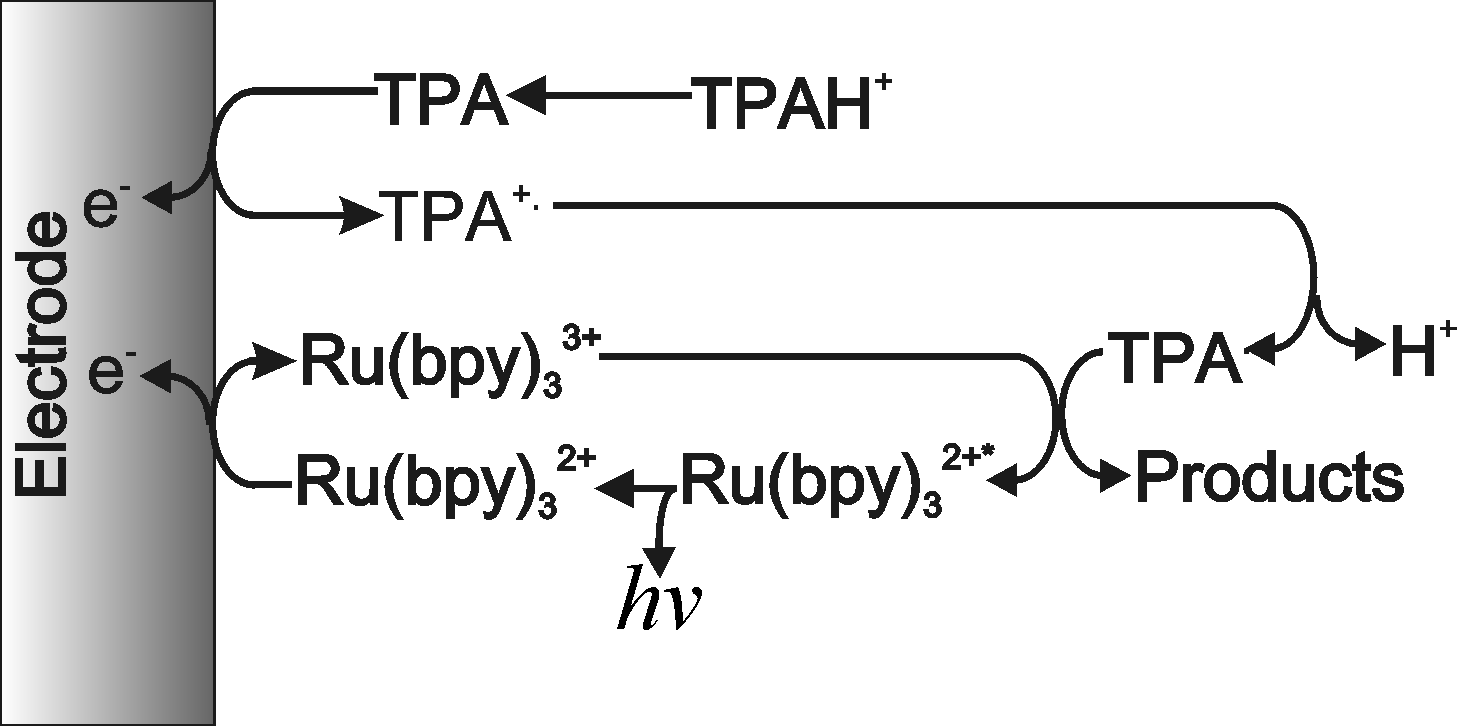

Supplement: Scheme S2 — The schematic electrochemiluminescence mechanism of TPA in Ru(bpy)3 2+-modified electrode [2], [3]. (1.06 MB TIF) [file pone.0006451.s006.tif]
